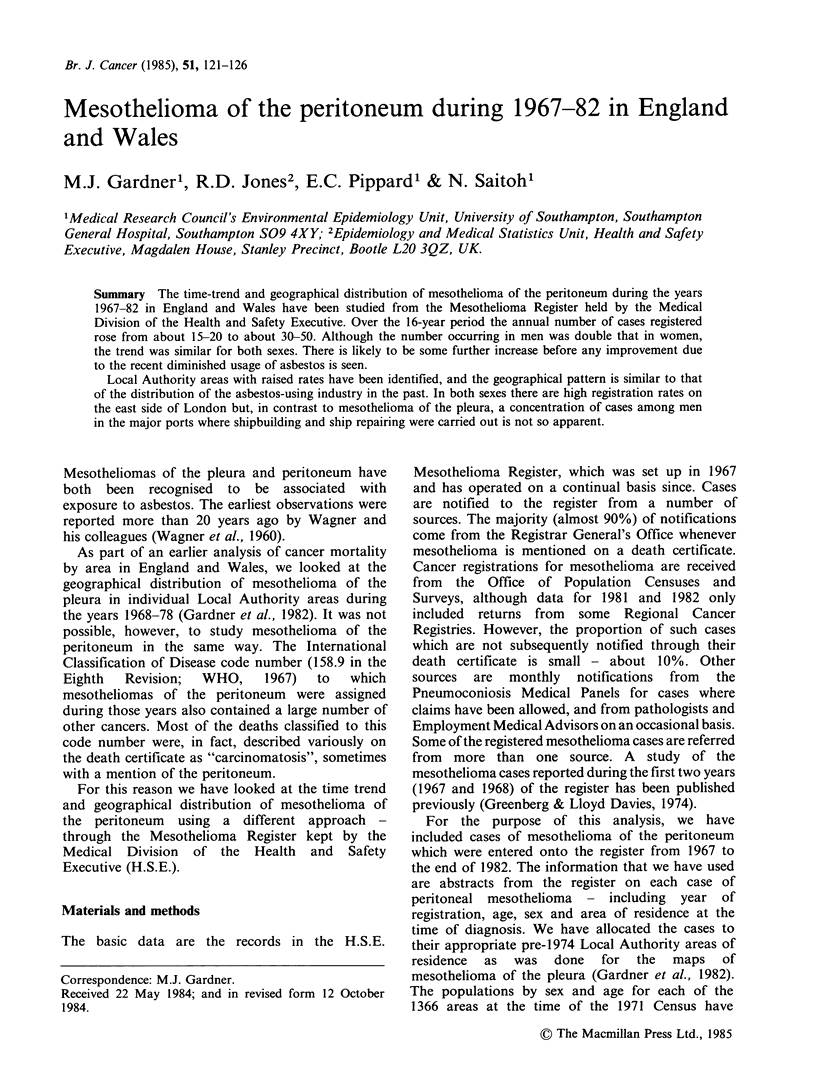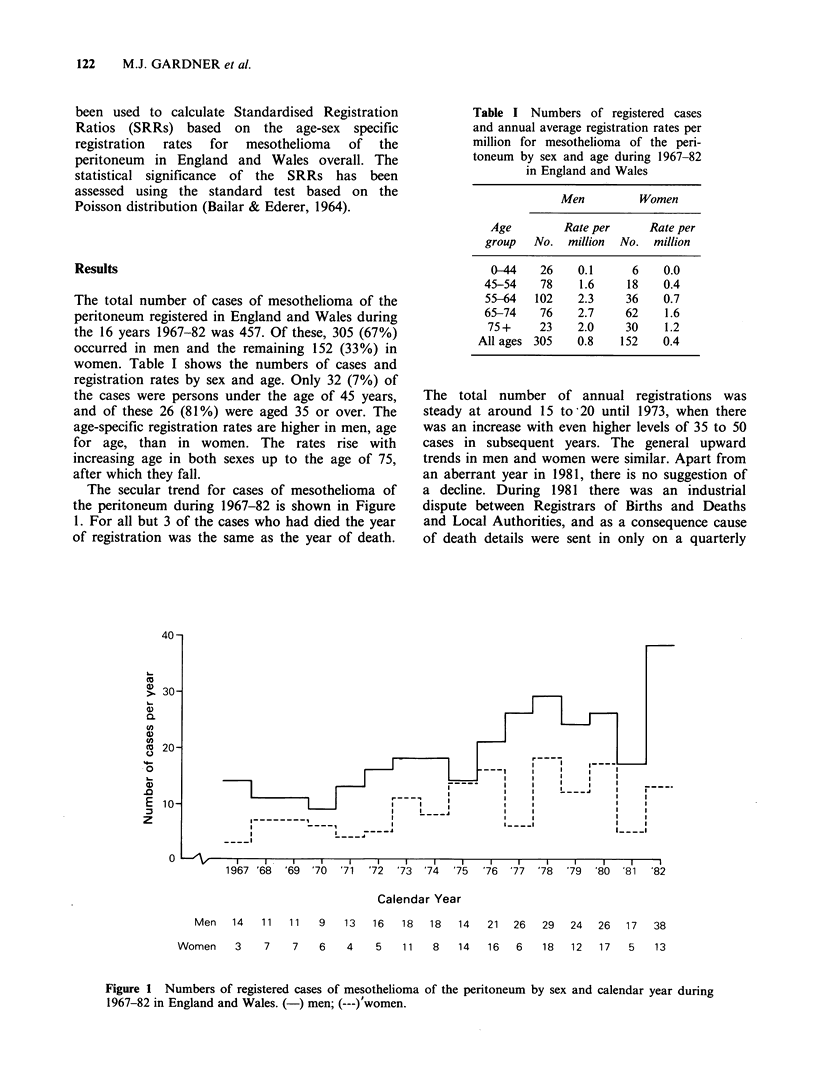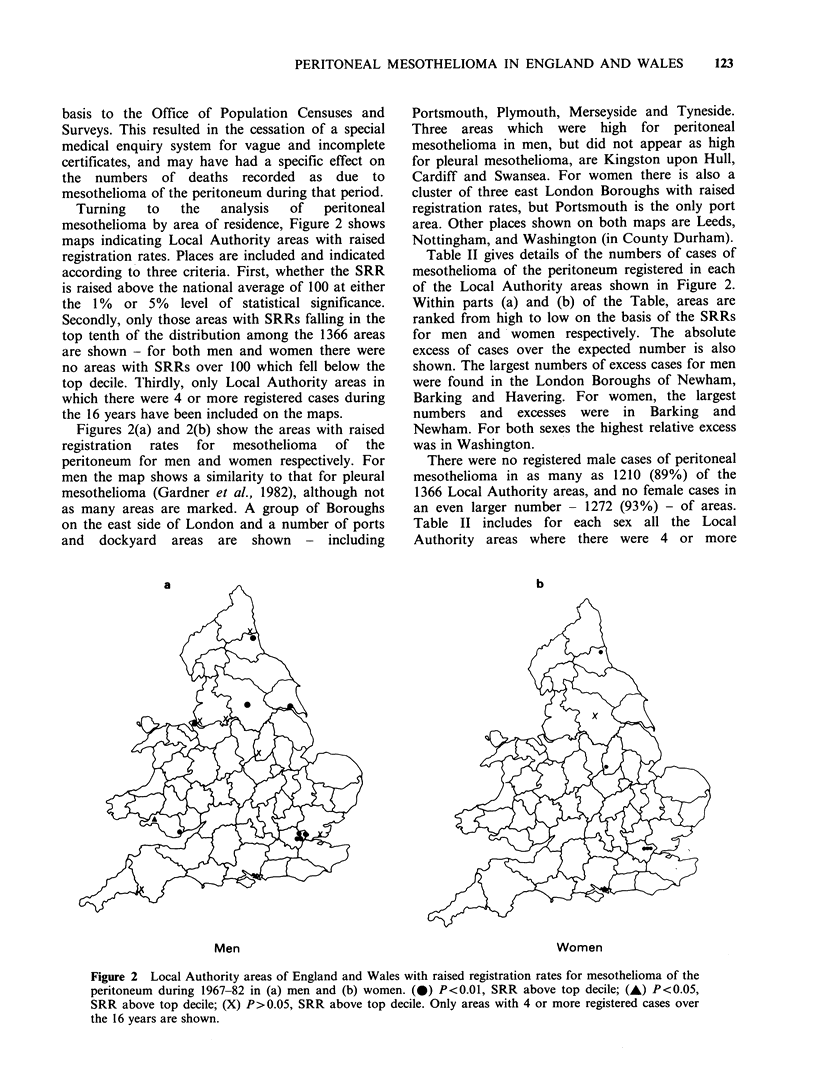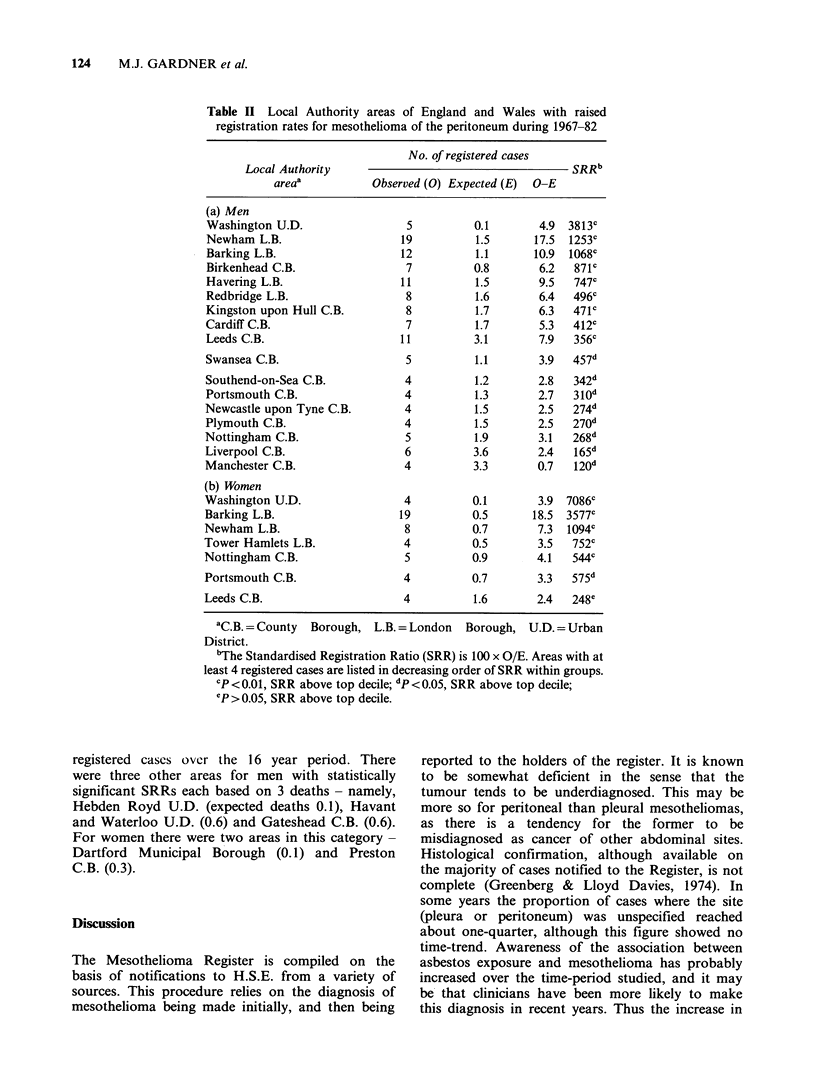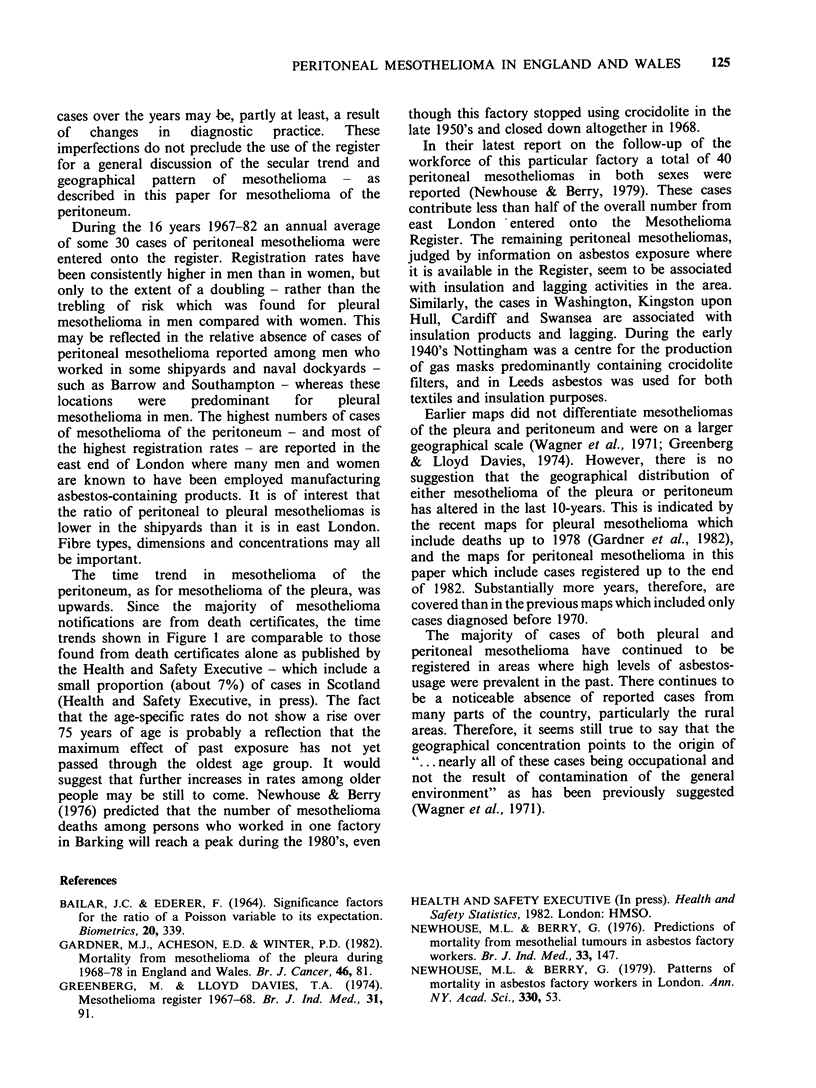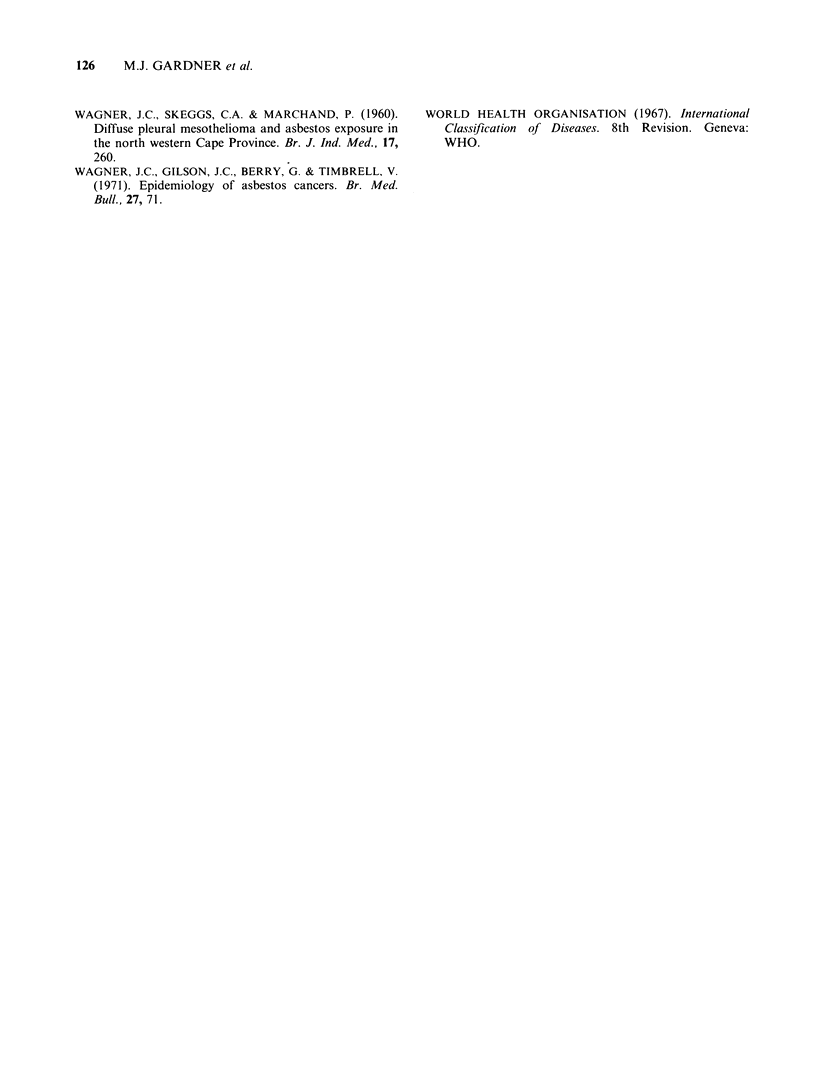# Mesothelioma of the peritoneum during 1967-82 in England and Wales.

**DOI:** 10.1038/bjc.1985.17

**Published:** 1985-01

**Authors:** M. J. Gardner, R. D. Jones, E. C. Pippard, N. Saitoh

## Abstract

The time-trend and geographical distribution of mesothelioma of the peritoneum during the years 1967-82 in England and Wales have been studied from the Mesothelioma Register held by the Medical Division of the Health and Safety Executive. Over the 16-year period the annual number of cases registered rose from about 15-20 to about 30-50. Although the number occurring in men was double that in women, the trend was similar for both sexes. There is likely to be some further increase before any improvement due to the recent diminished usage of asbestos is seen. Local Authority areas with raised rates have been identified, and the geographical pattern is similar to that of the distribution of the asbestos-using industry in the past. In both sexes there are high registration rates on the east side of London but, in contrast to mesothelioma of the pleura, a concentration of cases among men in the major ports where shipbuilding and ship repairing were carried out is not so apparent.


Br. J. Cancer (1985), 51, 121–126

### Mesothelioma of the peritoneum during 1967–82 in England and Wales

M.J. Gardner[1], R.D. Jones[2], E.C. Pippard[1] & N. Saitoh[1]

[1]*Medical Research Council's Environmental Epidemiology Unit, University of Southampton, Southampton General Hospital, Southampton SO9 4XY;* [2]*Epidemiology and Medical Statistics Unit, Health and Safety Executive, Magdalen House, Stanley Precinct, Bootle L20 3QZ, UK.*

**Summary** The time-trend and geographical distribution of mesothelioma of the peritoneum during the years 1967–82 in England and Wales have been studied from the Mesothelioma Register held by the Medical Division of the Health and Safety Executive. Over the 16-year period the annual number of cases registered rose from about 15–20 to about 30–50. Although the number occurring in men was double that in women, the trend was similar for both sexes. There is likely to be some further increase before any improvement due to the recent diminished usage of asbestos is seen.

Local Authority areas with raised rates have been identified, and the geographical pattern is similar to that of the distribution of the asbestos-using industry in the past. In both sexes there are high registration rates on the east side of London but, in contrast to mesothelioma of the pleura, a concentration of cases among men in the major ports where shipbuilding and ship repairing were carried out is not so apparent.

Mesotheliomas of the pleura and peritoneum have both been recognised to be associated with exposure to asbestos. The earliest observations were reported more than 20 years ago by Wagner and his colleagues (Wagner *et al.*, 1960).

As part of an earlier analysis of cancer mortality by area in England and Wales, we looked at the geographical distribution of mesothelioma of the pleura in individual Local Authority areas during the years 1968–78 (Gardner *et al.*, 1982). It was not possible, however, to study mesothelioma of the peritoneum in the same way. The International Classification of Disease code number (158.9 in the Eighth Revision; WHO, 1967) to which mesotheliomas of the peritoneum were assigned during those years also contained a large number of other cancers. Most of the deaths classified to this code number were, in fact, described variously on the death certificate as "carcinomatosis", sometimes with a mention of the peritoneum.

For this reason we have looked at the time trend and geographical distribution of mesothelioma of the peritoneum using a different approach – through the Mesothelioma Register kept by the Medical Division of the Health and Safety Executive (H.S.E.).

### Materials and methods

The basic data are the records in the H.S.E. Mesothelioma Register, which was set up in 1967 and has operated on a continual basis since. Cases are notified to the register from a number of sources. The majority (almost 90%) of notifications come from the Registrar General's Office whenever mesothelioma is mentioned on a death certificate. Cancer registrations for mesothelioma are received from the Office of Population Censuses and Surveys, although data for 1981 and 1982 only included returns from some Regional Cancer Registries. However, the proportion of such cases which are not subsequently notified through their death certificate is small – about 10%. Other sources are monthly notifications from the Pneumoconiosis Medical Panels for cases where claims have been allowed, and from pathologists and Employment Medical Advisors on an occasional basis. Some of the registered mesothelioma cases are referred from more than one source. A study of the mesothelioma cases reported during the first two years (1967 and 1968) of the register has been published previously (Greenberg & Lloyd Davies, 1974).

For the purpose of this analysis, we have included cases of mesothelioma of the peritoneum which were entered onto the register from 1967 to the end of 1982. The information that we have used are abstracts from the register on each case of peritoneal mesothelioma – including year of registration, age, sex and area of residence at the time of diagnosis. We have allocated the cases to their appropriate pre-1974 Local Authority areas of residence as was done for the maps of mesothelioma of the pleura (Gardner *et al.*, 1982). The populations by sex and age for each of the 1366 areas at the time of the 1971 Census have

Correspondence: M.J. Gardner.
Received 22 May 1984; and in revised form 12 October 1984.

© The Macmillan Press Ltd., 1985

been used to calculate Standardised Registration Ratios (SRRs) based on the age-sex specific registration rates for mesothelioma of the peritoneum in England and Wales overall. The statistical significance of the SRRs has been assessed using the standard test based on the Poisson distribution (Bailar & Ederer, 1964).

### Results

The total number of cases of mesothelioma of the peritoneum registered in England and Wales during the 16 years 1967–82 was 457. Of these, 305 (67%) occurred in men and the remaining 152 (33%) in women. Table I shows the numbers of cases and registration rates by sex and age. Only 32 (7%) of the cases were persons under the age of 45 years, and of these 26 (81%) were aged 35 or over. The age-specific registration rates are higher in men, age for age, than in women. The rates rise with increasing age in both sexes up to the age of 75, after which they fall.

The secular trend for cases of mesothelioma of the peritoneum during 1967–82 is shown in Figure 1. For all but 3 of the cases who had died the year of registration was the same as the year of death.

**Table I** Numbers of registered cases and annual average registration rates per million for mesothelioma of the peritoneum by sex and age during 1967–82 in England and Wales

| | *Men* | | *Women* | |
|---|---|---|---|---|
| *Age group* | *No.* | *Rate per million* | *No.* | *Rate per million* |
| 0–44 | 26 | 0.1 | 6 | 0.0 |
| 45–54 | 78 | 1.6 | 18 | 0.4 |
| 55–64 | 102 | 2.3 | 36 | 0.7 |
| 65–74 | 76 | 2.7 | 62 | 1.6 |
| 75+ | 23 | 2.0 | 30 | 1.2 |
| All ages | 305 | 0.8 | 152 | 0.4 |

The total number of annual registrations was steady at around 15 to 20 until 1973, when there was an increase with even higher levels of 35 to 50 cases in subsequent years. The general upward trends in men and women were similar. Apart from an aberrant year in 1981, there is no suggestion of a decline. During 1981 there was an industrial dispute between Registrars of Births and Deaths and Local Authorities, and as a consequence cause of death details were sent in only on a quarterly

**Figure 1** Numbers of registered cases of mesothelioma of the peritoneum by sex and calendar year during 1967–82 in England and Wales. (—) men; (---) women.

basis to the Office of Population Censuses and Surveys. This resulted in the cessation of a special medical enquiry system for vague and incomplete certificates, and may have had a specific effect on the numbers of deaths recorded as due to mesothelioma of the peritoneum during that period.

Turning to the analysis of peritoneal mesothelioma by area of residence, Figure 2 shows maps indicating Local Authority areas with raised registration rates. Places are included and indicated according to three criteria. First, whether the SRR is raised above the national average of 100 at either the 1% or 5% level of statistical significance. Secondly, only those areas with SRRs falling in the top tenth of the distribution among the 1366 areas are shown – for both men and women there were no areas with SRRs over 100 which fell below the top decile. Thirdly, only Local Authority areas in which there were 4 or more registered cases during the 16 years have been included on the maps.

Figures 2(a) and 2(b) show the areas with raised registration rates for mesothelioma of the peritoneum for men and women respectively. For men the map shows a similarity to that for pleural mesothelioma (Gardner *et al.*, 1982), although not as many areas are marked. A group of Boroughs on the east side of London and a number of ports and dockyard areas are shown – including Portsmouth, Plymouth, Merseyside and Tyneside. Three areas which were high for peritoneal mesothelioma in men, but did not appear as high for pleural mesothelioma, are Kingston upon Hull, Cardiff and Swansea. For women there is also a cluster of three east London Boroughs with raised registration rates, but Portsmouth is the only port area. Other places shown on both maps are Leeds, Nottingham, and Washington (in County Durham).

Table II gives details of the numbers of cases of mesothelioma of the peritoneum registered in each of the Local Authority areas shown in Figure 2. Within parts (a) and (b) of the Table, areas are ranked from high to low on the basis of the SRRs for men and women respectively. The absolute excess of cases over the expected number is also shown. The largest numbers of excess cases for men were found in the London Boroughs of Newham, Barking and Havering. For women, the largest numbers and excesses were in Barking and Newham. For both sexes the highest relative excess was in Washington.

There were no registered male cases of peritoneal mesothelioma in as many as 1210 (89%) of the 1366 Local Authority areas, and no female cases in an even larger number – 1272 (93%) – of areas. Table II includes for each sex all the Local Authority areas where there were 4 or more

**Figure 2** Local Authority areas of England and Wales with raised registration rates for mesothelioma of the peritoneum during 1967–82 in (a) men and (b) women. (●) $P<0.01$, SRR above top decile; (▲) $P<0.05$, SRR above top decile; (X) $P>0.05$, SRR above top decile. Only areas with 4 or more registered cases over the 16 years are shown.

**Table II** Local Authority areas of England and Wales with raised registration rates for mesothelioma of the peritoneum during 1967–82

| *Local Authority area*[a] | *No. of registered cases* | | | *SRR*[b] |
|---|---|---|---|---|
| | *Observed (O)* | *Expected (E)* | *O–E* | |
| (a) *Men* | | | | |
| Washington U.D. | 5 | 0.1 | 4.9 | 3813[c] |
| Newham L.B. | 19 | 1.5 | 17.5 | 1253[c] |
| Barking L.B. | 12 | 1.1 | 10.9 | 1068[c] |
| Birkenhead C.B. | 7 | 0.8 | 6.2 | 871[c] |
| Havering L.B. | 11 | 1.5 | 9.5 | 747[c] |
| Redbridge L.B. | 8 | 1.6 | 6.4 | 496[c] |
| Kingston upon Hull C.B. | 8 | 1.7 | 6.3 | 471[c] |
| Cardiff C.B. | 7 | 1.7 | 5.3 | 412[c] |
| Leeds C.B. | 11 | 3.1 | 7.9 | 356[c] |
| Swansea C.B. | 5 | 1.1 | 3.9 | 457[d] |
| Southend-on-Sea C.B. | 4 | 1.2 | 2.8 | 342[d] |
| Portsmouth C.B. | 4 | 1.3 | 2.7 | 310[d] |
| Newcastle upon Tyne C.B. | 4 | 1.5 | 2.5 | 274[d] |
| Plymouth C.B. | 4 | 1.5 | 2.5 | 270[d] |
| Nottingham C.B. | 5 | 1.9 | 3.1 | 268[d] |
| Liverpool C.B. | 6 | 3.6 | 2.4 | 165[d] |
| Manchester C.B. | 4 | 3.3 | 0.7 | 120[d] |
| (b) *Women* | | | | |
| Washington U.D. | 4 | 0.1 | 3.9 | 7086[c] |
| Barking L.B. | 19 | 0.5 | 18.5 | 3577[c] |
| Newham L.B. | 8 | 0.7 | 7.3 | 1094[c] |
| Tower Hamlets L.B. | 4 | 0.5 | 3.5 | 752[c] |
| Nottingham C.B. | 5 | 0.9 | 4.1 | 544[c] |
| Portsmouth C.B. | 4 | 0.7 | 3.3 | 575[d] |
| Leeds C.B. | 4 | 1.6 | 2.4 | 248[e] |

[a]C.B. = County Borough, L.B. = London Borough, U.D. = Urban District.
[b]The Standardised Registration Ratio (SRR) is 100 × O/E. Areas with at least 4 registered cases are listed in decreasing order of SRR within groups.
[c]$P < 0.01$, SRR above top decile; [d]$P < 0.05$, SRR above top decile;
[e]$P > 0.05$, SRR above top decile.

registered cases over the 16 year period. There were three other areas for men with statistically significant SRRs each based on 3 deaths – namely, Hebden Royd U.D. (expected deaths 0.1), Havant and Waterloo U.D. (0.6) and Gateshead C.B. (0.6). For women there were two areas in this category – Dartford Municipal Borough (0.1) and Preston C.B. (0.3).

### Discussion

The Mesothelioma Register is compiled on the basis of notifications to H.S.E. from a variety of sources. This procedure relies on the diagnosis of mesothelioma being made initially, and then being reported to the holders of the register. It is known to be somewhat deficient in the sense that the tumour tends to be underdiagnosed. This may be more so for peritoneal than pleural mesotheliomas, as there is a tendency for the former to be misdiagnosed as cancer of other abdominal sites. Histological confirmation, although available on the majority of cases notified to the Register, is not complete (Greenberg & Lloyd Davies, 1974). In some years the proportion of cases where the site (pleura or peritoneum) was unspecified reached about one-quarter, although this figure showed no time-trend. Awareness of the association between asbestos exposure and mesothelioma has probably increased over the time-period studied, and it may be that clinicians have been more likely to make this diagnosis in recent years. Thus the increase in cases over the years may be, partly at least, a result of changes in diagnostic practice. These imperfections do not preclude the use of the register for a general discussion of the secular trend and geographical pattern of mesothelioma – as described in this paper for mesothelioma of the peritoneum.

During the 16 years 1967–82 an annual average of some 30 cases of peritoneal mesothelioma were entered onto the register. Registration rates have been consistently higher in men than in women, but only to the extent of a doubling – rather than the trebling of risk which was found for pleural mesothelioma in men compared with women. This may be reflected in the relative absence of cases of peritoneal mesothelioma reported among men who worked in some shipyards and naval dockyards – such as Barrow and Southampton – whereas these locations were predominant for pleural mesothelioma in men. The highest numbers of cases of mesothelioma of the peritoneum – and most of the highest registration rates – are reported in the east end of London where many men and women are known to have been employed manufacturing asbestos-containing products. It is of interest that the ratio of peritoneal to pleural mesotheliomas is lower in the shipyards than it is in east London. Fibre types, dimensions and concentrations may all be important.

The time trend in mesothelioma of the peritoneum, as for mesothelioma of the pleura, was upwards. Since the majority of mesothelioma notifications are from death certificates, the time trends shown in Figure 1 are comparable to those found from death certificates alone as published by the Health and Safety Executive – which include a small proportion (about 7%) of cases in Scotland (Health and Safety Executive, in press). The fact that the age-specific rates do not show a rise over 75 years of age is probably a reflection that the maximum effect of past exposure has not yet passed through the oldest age group. It would suggest that further increases in rates among older people may be still to come. Newhouse & Berry (1976) predicted that the number of mesothelioma deaths among persons who worked in one factory in Barking will reach a peak during the 1980's, even though this factory stopped using crocidolite in the late 1950's and closed down altogether in 1968.

In their latest report on the follow-up of the workforce of this particular factory a total of 40 peritoneal mesotheliomas in both sexes were reported (Newhouse & Berry, 1979). These cases contribute less than half of the overall number from east London entered onto the Mesothelioma Register. The remaining peritoneal mesotheliomas, judged by information on asbestos exposure where it is available in the Register, seem to be associated with insulation and lagging activities in the area. Similarly, the cases in Washington, Kingston upon Hull, Cardiff and Swansea are associated with insulation products and lagging. During the early 1940's Nottingham was a centre for the production of gas masks predominantly containing crocidolite filters, and in Leeds asbestos was used for both textiles and insulation purposes.

Earlier maps did not differentiate mesotheliomas of the pleura and peritoneum and were on a larger geographical scale (Wagner *et al.*, 1971; Greenberg & Lloyd Davies, 1974). However, there is no suggestion that the geographical distribution of either mesothelioma of the pleura or peritoneum has altered in the last 10-years. This is indicated by the recent maps for pleural mesothelioma which include deaths up to 1978 (Gardner *et al.*, 1982), and the maps for peritoneal mesothelioma in this paper which include cases registered up to the end of 1982. Substantially more years, therefore, are covered than in the previous maps which included only cases diagnosed before 1970.

The majority of cases of both pleural and peritoneal mesothelioma have continued to be registered in areas where high levels of asbestos-usage were prevalent in the past. There continues to be a noticeable absence of reported cases from many parts of the country, particularly the rural areas. Therefore, it seems still true to say that the geographical concentration points to the origin of "...nearly all of these cases being occupational and not the result of contamination of the general environment" as has been previously suggested (Wagner *et al.*, 1971).

### References

BAILAR, J.C. & EDERER, F. (1964). Significance factors for the ratio of a Poisson variable to its expectation. *Biometrics*, **20**, 339.

GARDNER, M.J., ACHESON, E.D. & WINTER, P.D. (1982). Mortality from mesothelioma of the pleura during 1968–78 in England and Wales. *Br. J. Cancer*, **46**, 81.

GREENBERG, M. & LLOYD DAVIES, T.A. (1974). Mesothelioma register 1967–68. *Br. J. Ind. Med.*, **31**, 91.

HEALTH AND SAFETY EXECUTIVE (In press). *Health and Safety Statistics*, 1982. London: HMSO.

NEWHOUSE, M.L. & BERRY, G. (1976). Predictions of mortality from mesothelial tumours in asbestos factory workers. *Br. J. Ind. Med.*, **33**, 147.

NEWHOUSE, M.L. & BERRY, G. (1979). Patterns of mortality in asbestos factory workers in London. *Ann. NY. Acad. Sci.*, **330**, 53.

WAGNER, J.C., SKEGGS, C.A. & MARCHAND, P. (1960). Diffuse pleural mesothelioma and asbestos exposure in the north western Cape Province. *Br. J. Ind. Med.*, **17**, 260.

WAGNER, J.C., GILSON, J.C., BERRY, G. & TIMBRELL, V. (1971). Epidemiology of asbestos cancers. *Br. Med. Bull.*, **27**, 71.

WORLD HEALTH ORGANISATION (1967). *International Classification of Diseases*. 8th Revision. Geneva: WHO.